# Higher Inflammation Is Associated with Cardiometabolic Phenotype and Biochemical Health in Women with Obesity

**DOI:** 10.1159/000522564

**Published:** 2022-03-18

**Authors:** Sarah Louise Killeen, David F. Byrne, Aisling A. Geraghty, Mark T. Kilbane, Patrick J. Twomey, Malachi J. McKenna, Cara A. Yelverton, Radka Saldova, Douwe Van Sinderen, Paul D. Cotter, Eileen F. Murphy, Fionnuala M. McAuliffe

**Affiliations:** ^a^UCD Perinatal Research Centre, School of Medicine, University College Dublin, National Maternity Hospital, Dublin, Ireland; ^b^Department of Clinical Chemistry, St Vincent's University Hospital, Dublin, Ireland; ^c^The National Institute for Bioprocessing, Research, and Training (NIBRT), Dublin, Ireland; ^d^UCD School of Medicine, College of Health and Agricultural Science (CHAS), University College Dublin (UCD), Dublin, Ireland; ^e^APC Microbiome Ireland, National University of Ireland, Cork, Ireland; ^f^School of Microbiology, National University of Ireland, Cork, Ireland; ^g^Teagasc Food Research Centre, Moorepark, Cork, Ireland; ^h^PrecisionBiotics Group Ltd., Cork Airport Business Park, Cork, Ireland

**Keywords:** Obesity, Metabolic health, Women's health, Inflammation

## Abstract

**Introduction:**

Metabolic or inflammatory markers may predict adverse outcomes in women with obesity. We sought to describe metabolic-obesity phenotypes of women using novel staging tools and investigate relationships with inflammation.

**Methods:**

In a cross-sectional study, we collected fasting blood samples from sixty-four females with body mass index (BMI) ≥28 kg/m<sup>2</sup>. Participants were classified as metabolically healthy or metabolically unhealthy obesity (MUO) using the cardiometabolic disease staging system (CMDS) and Edmonton obesity staging system (EOSS). Data were analyzed using independent sample *t* tests, Pearson's correlations, and multiple logistic regression.

**Results:**

Mean (SD) age was 40.2 (9.3) years with median (IQR) BMI 31.8 (30.3–35.7) kg/m<sup>2</sup>. The prevalence of MUO was 46.9% and 81.3% using CMDS and EOSS criteria, respectively. Women with raised CMDS scores had higher C3 (1.34 [0.20] vs. 1.18 [0.15], *p* = 0.001) and C-reactive protein (CRP) (2.89 [1.31–7.61] vs. 1.39 [0.74–3.60], *p* = 0.034). C3 correlated with insulin (*r* = 0.52), hemoglobin A1c (*r* = 0.37), and C-peptide (*r* = 0.58), all *p* < 0.05. C3 above the median (>1.23 g/L) increased odds of raised CMDS score, when controlled for age, BMI, ethnicity, and smoking (OR = 6.56, 95% CI: 1.63, 26.47, *p* = 0.008).

**Conclusion:**

The prevalence of MUO was lower using CMDS than EOSS. C3 and CRP may be useful clinical biomarkers of risk or treatment targets in women with obesity.

## Introduction

Maternal obesity is associated with an increased risk of adverse maternal and fetal outcomes [1]. The prevalence of obesity in women of reproductive age is rising in both low-middle- and high-income countries, becoming the predominant presentation in antenatal services. In 2018, prepregnancy overweight or obesity prevalence was 42% in the USA, 30% in Europe, and 10% in Asia [2]. Rather than using body mass index (BMI), maternal risk could be defined using other markers. Prepregnancy metabolic markers can predict pregnancy outcomes such as gestational diabetes and preeclampsia [3, 4, 5]. The cardiometabolic disease staging system (CMDS) and Edmonton obesity staging system (EOSS) are two clinical scoring systems, both considering metabolic health, that measure obesity severity [6]. Compared to BMI, evidence suggests the EOSS can better predict health service usage and treatment outcomes [7]. Ejima et al. [6], using data from the National Health and Nutrition Examination Survey 2014, compared CMDS and EOSS on the prediction of mortality. They found CMDS, which uses fewer criteria, had greater discriminatory value [6]. In addition, novel inflammatory risk factors such as C3 complement protein (C3) or C-reactive protein (CRP) have been proposed to identify potential cardiometabolic risk [8, 9, 10]. Karelis et al. [11] found that postmenopausal women with metabolically healthy obesity (MHO) had lower CRP than those with metabolically unhealthy obesity (MUO). In an Irish study of men and women aged 45–74 years, lower C3 increased odds of MHO [12]. The application of the CMDS and EOSS has never been compared in women with obesity of childbearing age. It is also unclear if there is a relationship between inflammatory markers CRP and C3 and MHO in this group. The aim of this study was to determine the metabolic-obesity phenotypes of women of reproductive age using the CMDS and EOSS and explore associations with inflammation (C3 and CRP).

## Materials and Methods

### Study Design

Data for this cross-sectional study came from baseline information collected from women as part of the screening visit for the GetGutsy study (ISRCTN11295995). The GetGutsy study was a double-blinded randomized control trial of a probiotic versus placebo capsule in nonpregnant women with overweight or obesity. This was a single-center study carried out from September 2018 to January 2020 at the UCD Perinatal Research Centre. The center is affiliated with the National Maternity Hospital, a tertiary University Hospital for maternity services in Dublin, and University College Dublin, Ireland. Institutional ethical approval was granted by University College Dublin and the National Maternity Hospital in 2017 (EC 28.2017) and updated in 2019 (EC 28.2017). Written informed consent was obtained from participants to participate in the study. Participants self-identified to the research team in response to traditional and digital recruitment strategies. General eligibility was assessed through self-reported data received by phone or email. Potentially eligible women were invited to an in-person screening visit, at which, self-reported data was confirmed. BMI was calculated by dividing the weight in kilograms by the height in meters squared.

The primary aim of the trial was the impact of a probiotic on high-sensitivity CRP in women with overweight or obesity and deranged lipid profiles, but no established cardiometabolic disease. Women were eligible for inclusion in the trial if they were aged 18–65 years, English speaking, not pregnant, lactating or planning a pregnancy in the next 3 months, had a BMI ≥28 kg/m^2^, and were not planning to lose weight or change their lifestyle in the next 3 months. The latter criteria were chosen so that the unique effect of the probiotic on inflammation could be assessed. Women with a known history of cardiometabolic diseases were excluded from the study. Participants who met these criteria were provided a fasting blood sample to assess lipid profiles, taken by women were randomized to receive the probiotic only if they had an atherogenic lipid profile using criteria of the American College of Cardiology (high-density lipoprotein [HDL] cholesterol <1.29 mmol/L and/or triglyceride ≥1.7 mmol/L) [13]. This is based on the expected mechanism of action of the probiotic which preliminary data suggest may be related to lipid metabolism.

Recruitment for the GetGutsy trial was stopped because insufficient numbers of women, who met all the inclusion criteria and resided within a reasonable distance of this single-center trial, were identified within the funded study period. Due to the breath of valuable data collected as part of the screening process, we share this data as a cross-sectional analysis. The sample size includes 64 women for which serum was available. All data were collected prior to randomization and included women with and without an atherogenic lipid profile.

### Biochemical Analyses

Blood samples taken into serum tubes were centrifuged at 3,000 rpm for 10 min. Samples were stored in the vacutainers at 4°C as soon as possible after venipuncture and once centrifuged, the aliquots were stored at −80°C pending analysis. Serum samples were used to measure C3, CRP, glucose, total cholesterol, HDL cholesterol, and triglycerides. Analyses were performed on a Roche Cobas 8000 automated chemistry system. Serum measurements of insulin and C-peptide were determined using the Cobas Roche e602 immunoassay system. Low-density lipoprotein (LDL) cholesterol was estimated using the equation of Friedewald et al. [14]. Ethylenediaminetetraacetic acid samples were available for 37 participants. Hemoglobin A1c was analyzed in whole blood Ethylenediaminetetraacetic acid samples using the Menarini/ARKRAY ADAMS^TM^ A1C HA-8180V system.

### Metabolic Phenotype

Cardiometabolic markers were used to classify women as MHO or MUO, separately using the EOSS and CMDS. For this study, we selected the biochemical cutoffs used by Canning et al. [15]. Women were given an EOSS score ≥1 if they met any of the following criteria: fasting glucose ≥5.6 mmol/L, total cholesterol ≥5.2 mmol/L, LDL cholesterol >3.3 mmol/L, HDL cholesterol <1.6 mmol/L, and triglyceride ≥1.7 mmol/L. Higher EOSS score indicates greater metabolic derangement. An EOSS score of zero was assigned if all the cardiometabolic markers were within the cutoffs. We applied the CMDS in a similar way to the EOSS; however, the CMDS includes fewer markers and includes female-specific cutoffs [16]. We did not apply high-risk or end-stage criteria (EOSS/CMDS stages 3 and 4) to this cohort as the presence of known conditions such as cardiovascular disease or type 2 diabetes were exclusion criteria for the study. To create MHO and MUO groups, dichotomous categorical variables were generated for CMDS (score ≥1 yes/no) and EOSS (score ≥1 yes/no).

### Statistical Analysis

Categorical variables are presented as number and frequency (%). Continuous variables were assessed for normality through visual inspection of histograms, the Shapiro-Wilk test for normality, and the inspection of descriptive data. All skewed data were log_10_ transformed prior to analysis. Continuous variables are presented as mean (standard deviation) or median and interquartile range (25th, 75th centile). Comparison statistics were generated for the entire group and based on gravidity, through independent sample *t* tests. Chi-square (χ^2^) tests were used to compare categorical variables except when expected values in the 2 × 2 table were <5 when Fisher's exact was used. Bivariate associations were tested using Pearson's product-moment correlations between both C3 and CRP with cardiometabolic markers. Single variable binary logistic regression was completed for C3, CRP, and potential confounders, predicting CMDS score. Multiple logistic regression was used to identify the predictive value of C3 and CRP with metabolic-obesity phenotype while controlling for confounders (age, BMI, ethnicity [Caucasian yes/no], and smoking [current smoker yes/no]). Variables were included in the regression if the Wald statistic *p* value was <0.25. Statistical analysis was performed using IBM SPSS software for Windows, version 24.0 (SPSS Inc., Chicago, IL, USA). Significance was determined at *p* < 0.05. All analyses were done with pairwise deletion of missing variables. PS: Power and Sample Size Calculation version 3.1.6 was used retrospectively to determine the power to detect a difference in mean C3 between MHO (CMDS 0) and unhealthy (CMDS ≥1), following the method of DuPont and Plummer [17].

## Results

### General Characteristics

Demographics and cardiometabolic parameters are presented in Table 1. Mean age was 40.16 (9.31) years. Over a quarter (29.7%) was below 35 years of age. Nearly all women (95.1%) had completed some third-level education (Table 1). Data on gravidity were available for 36 women. Over half (63.9%) had been pregnant before and there was no difference in C3 (1.14 [0.15] vs. 1.23 [0.16] g/L, *p* = 0.099) or CRP (2.32 [2.75] vs. 3.59 [3.36] mg/L, *p* = 0.165) between first time mothers versus previously pregnant women. In χ^2^ tests, the proportion of women meeting MUO criteria using CMDS or EOSS did not differ based on pregnancy history (*p* > 0.05).

Just under half (46.9%) of women were classified as MUO using the CMDS criteria and the majority (81.3%) of women were classified as MUO using EOSS. Taking each cardiometabolic marker separately, 8 (12.5%) had a glucose concentration ≥5.6 mmol/L, 18 (26.9%) had HDL cholesterol levels <1.29 mmol/L, 44 (68.8%) had HDL cholesterol <1.6 mmol/L, 15 (23.4%) had LDL cholesterol >3.3 mmol/L, 16 (25.0%) had total cholesterol ≥5.2 mmol/L, and 13 (20.3%) had a triglyceride level ≥1.7 mmol/L. When we looked at individual CRP values in relation to clinical cutoffs, 67.2% had a CRP value above 1 mg/L and 50.6% had a CRP above 3 mg/L. The proportion of women with a CRP level above the median was not different based on EOSS (χ^2^ (1, *n* = 64) = 0.58, *p* = 1.00) or CMDS (χ^2^ (1, *n* = 64) = 0.95, *p* = 0.452). The proportion of women with a C3 level above the median, however, was greater in MUO groups using both EOSS (χ^2^ (1, *n* = 64) = 5.41, *p* = 0.020) and CMDS (χ^2^ (1, *n* = 64) = 8.88, *p* = 0.003). Mean C3 was lower in the MHO group using CMDS cutoffs (1.18 [0.15] vs. 1.34 [0.20], *p* = 0.001, 1-β of 94.6%).

### Metabolic Phenotype and Inflammation

There were significant associations between C3 and CRP with markers of lipid metabolism, and glycemic control (Table 2). Where both C3 and CRP were significantly associated with health markers, *r*^2^ values were higher for C3 in all cases, explaining 26.5% of the variance in insulin and 33.2% C-peptide. As a result, we developed a model in multiple logistic regression to determine the relationship between C3, CRP, and CMDS phenotypes. In the final model, which controlled for age, BMI, ethnicity, and smoking, having a C3 value above the median resulted in increased odds of MUO using CMDS (odds ratio [95% CI] = 6.56 [1.63, 26.47], *p* = 0.008) while value for CRP was nonsignificant (*p* = 0.797).

## Discussion

In this study, most participants had MUO using EOSS and 46.9% using CMDS criteria. Women with MUO had higher inflammation, insulin, C-peptide, and hemoglobin A1c, compared to those with MHO. We found that inflammation was a significant driver of the variance in cardiometabolic and glycemic markers. In comparison to CRP, C3 contributed a greater proportion of the variance as measured by *r*^2^. In addition, having a C3 level above the median increased the odds of MUO, even when controlled for BMI.

Application of obesity staging systems may aid in decision-making around the medical needs of individuals with obesity. Recently, the concept of treatment prioritization based on metabolic factors was applied in relation to fertility care of women with obesity [18]. In this Canadian study, a “metabolic global approach” was taken whereby achievement of healthy metabolic indices was recommended before commencing treatment [18]. The Society of Obstetricians and Gynecologists of Canada recommend baseline screening for cholesterol and triglycerides as part of preconception care for women with obesity [19, 20]. Like previous studies, we found low HDL was the most common biochemical marker of metabolic risk and there is evidence to suggest that low HDL could be the first indicator of future metabolic ill health in younger adults [21]. The EOSS has a greater number of inclusion criteria and a higher cutoff for HDL (1.6 vs. 1.29 mmol/L in the CMDS). This resulted in a larger proportion of women with MUO, potentially limiting the utility of this in the clinical setting.

A study of individuals with prediabetes by Gopalan et al. [22] suggests that awareness of cardiometabolic status increases the likelihood of engaging in healthy behaviors. Using data from the 2011 to 2014 National Health and Nutrition Examination Survey, Tsai et al. [23] found that awareness of cardiometabolic risk was low among those ≥40 years of age, seen only in those CMDS stage 4. This is the highest level in the CMDS and represents individuals with diagnosed type 2 diabetes and/or cardiovascular disease [23]. Our analysis shows just under half of these generally healthy women (46.9%) had one or more risk factors for the metabolic syndrome as assessed by CMDS and most women had at least one metabolic risk factor from EOSS (81.3%). Testing and treatment of asymptomatic individuals for cardiovascular risk factors, including dyslipidemia has been shown to be cost-effective in some but not all studies and more research is needed [24, 25, 26]. Regardless, the American Heart Association (AHA) and the European guidelines on cardiovascular disease recommend generally healthy people are screened for cardiovascular risk, with the AHA suggesting this should start from 20 years of age [13, 27]. Testing and treatment of asymptomatic individuals for cardiovascular risk factors, including dyslipidemia has been shown to be cost-effective in some but not all studies and more research is needed [24, 25, 26]. Regardless, the AHA and the European guidelines on cardiovascular disease recommend generally healthy people are screened for cardiovascular risk, with the AHA suggesting this should start from 20 years of age [13, 27].

In this cross-sectional analysis, we found, C3 was the only marker associated with increased odds of MUO. In a study of the HELENA cohort, adolescent girls with MUO had higher C3 than MHO, and both groups had higher levels than those with lower BMI and CRP was not different between groups [28]. Higher levels of C3 have been found in women with insulin resistance compared to insulin-sensitive controls [29]. In pregnancy, relationships between increased C3 and insulin resistance and lipid profiles have also been reported [30]. In an elderly population, Muscari et al. [31], found that when compared to other inflammatory markers, C3 was most strongly associated with insulin resistance, after controlling for confounders. There is also longitudinal data suggesting the potential role of increased C3 in later cardiometabolic disease [32].

This is the first study to apply both the CMDS and EOSS to women of reproductive age with obesity, outside of pregnancy. Our selection of C3 adds to the novel yet growing literature on this protein in relation to adverse cardiometabolic outcomes. There are several limitations worth noting. Most women in our study were Caucasian, had third-level education, and were in employment. This should be considered when comparing more diverse cohorts. The sample size is small, and it is possible that some of the analyses lacked statistical power. The analyses are cross sectional, and this limits the ability to draw conclusions from the data. More longitudinal research is needed to confirm these findings in larger and more diverse populations before the results can be generalized for clinical practice.

## Conclusion

The proportion of women with MUO was lower with the CMDS compared to EOSS. Differences in inflammatory and glycemic markers were found between MHO and MUO, including C3. While more research is needed, our data suggest C3 may be a useful therapeutic target in clinical practice or a clinical marker of cardiometabolic risk in women with obesity.

## Statement of Ethics

This study protocol was reviewed and approved by University College Dublin and the National Maternity Hospital in 2017 (EC 28.2017) and updated in 2019 (EC 28.2017). Written informed consent was obtained from participants to participate in the study.

## Conflict of Interest Statement

The authors have no conflicts of interest to declare.

## Funding Sources

This publication has emanated from research supported in part by a research grant from Science Foundation Ireland (SFI) under Grant No. 12/RC/2273 and 16/SP/3827 and by a research grant from PrecisionBiotics Group Ltd.

## Author Contributions

All authors were involved in the conception and design of this study. S.L.K. conducted the analysis and wrote the manuscript with input from all other authors. All authors provided input into the study design, analytical methods, and revisions of the manuscript.

## Data Availability Statement

Data are available on request from the corresponding author.

## Figures and Tables

**Table 1:** Baseline demographics, inflammation, health markers, and metabolic-obesity phenotype

	Total, *n*		CMDS			EOSS		
			CMDS 0 (*n* = 34)	CDMS ≥ 1 (*n* = 30)	*p* value	EOSS 0 (*n =* 12)	EOSS ≥ 1 (*n* = 52)	*p* value
*Demographics*								
Age, years	63	40.16 (9.31)	41.44 (9.43)	38.66 (9.11)	0.240	41.42 (9.49)	39.86 (9.34)	0.607
BMI, kg/m^2^	64	31.82 (30.27–35.74) 31.56 (30.98,34.99) 33.02 (29.92,38.08) 0.133	32.71 (31.32,36.13) 31.68 (29.68,35.74)	0.766
Ethnicity (Caucasian), *n* (*%*)	64	58 (90.6)	33 (97.1)	25 (83.3)	0.090	12 (100)	48 (88.5)	0.584
Education (completed some third level), *n* (*%*)	61	58 (95.1)	32 (94.1)	26 (96.3)	>0.999	12 (100)	46 (93.9)	>0.999
Smoking (current), *n* (*%*)	64	9 (14.1)	3 (8.8)	6 (20.0)	0.285	2 (16.7)	7 (13.5)	0.672

*Health markers*								
C3, g/L	64	1.25 (0.19)	1.18 (0.15)	1.34 (0.20)	0.001	1.17 (0.16)	1.27 (0.20)	0.083
CRP, mg/L^a^	64	2.44 (0.90, 4.50)	1.39 (0.74, 3.60)	2.89 (1.31, 7.61)	0.034	2.68 (0.90, 4.66)	2.44 (0.89, 4.50)	0.832
Insulin, mU/L^a^	64	11.99 (8.74, 17.58)	10.88 (8.15, 14.41)	14.93 (10.63, 22.30) 0.007	9.31 (6.66, 13.29)	12.69 (9.11, 18.12)	0.037
C-peptide, µg/L^a^	64	2.63 (2.05, 3.26)	2.23 (1.80, 2.70)	2.96 (2.53, 3.68)	<0.001	1.93 (1.77, 2.87)	2.68 (2.18,3.53)	0.010
HbA1c, mmol/mol^b^	37	34.22 (2.78)	33.15 (1.75)	35.47 (3.26)	0.015	32.75 (1.40)	34.62 (2.95)	0.092

BMI, body mass index; CMDS, cardiometabolic disease staging system; CRP, C-reactive protein; C3, C3 complement protein; EOSS, Edmonton obesity staging system; HbA1c, Hemoglobin A1c; HDL, high-density lipoprotein; LDL, low-density lipoprotein. Values are presented as mean (standard deviation) for parametric data or median (interquartile range 25th, 75th) for nonparametric variables. *p* values from standard *t* tests comparing values grouped by metabolic-obesity phenotype. *p* values (two-tailed) for categorical variables were derived from Fisher's exact test in a 2 × 2 table.

a Denotes log transformed data were used in comparison statistic.

b HbA1c data available for *n* =37 women.

**Table 2:** Correlations among C3 Complement, CRP, and cardiometabolic markers (*n* = 64)

	CRP, mg/L			C3, g/L		
	*r*	*r* ^2^	*p* value	*r*	*r* ^2^	*p* value
CRP, mg/L^a^	−			0.624	0.389	<0.001
C3 complement, g/L	0.624	0.389	<0.001	−		
Total cholesterol, mmol/L^a^	−0.019	<0.001	0.883	−0.021	<0.001	0.868
HDL cholesterol, mmol/L^a^	−0.081	0.007	0.525	−0.242	0.059	0.054
LDL cholesterol, mmol/L	−0.112	0.013	0.376	−0.070	0.005	0.585
Triglyceride, mmol/L^a^	0.379	0.144	0.002	0.433	0.188	<0.001
Glucose, mmol/L	0.158	0.025	0.213	0.339	0.115	0.003
Insulin, mU/L^a^	0.285	0.081	0.023	0.515	0.265	<0.001
C-peptide, µg/L	0.401	0.161	0.001	0.576	0.332	<0.001
HbA1c, mmol/mol^b^	0.178	0.032	0.299	0.368	0.135	0.025

Values are Pearson's correlations. HbA1c, hemoglobin A1c; HDL, high-density lipoprotein; LDL, low-density lipoprotein; CRP, C-reactive protein; C3, C3 complement protein.

a Log transformed data were used.

b n = 38 for HbA1c.
